# Laser Atherectomy and Restenting of the Superficial Femoral Artery Using GORE VIABAHN Endoprosthesis Following Failure of Both Bare-Metal Stenting and Surgical Revascularization

**DOI:** 10.1155/2024/4950420

**Published:** 2024-11-05

**Authors:** Ahmed Khawer, Claro F. Diaz

**Affiliations:** ^1^DeBusk College of Osteopathic Medicine, Lincoln Memorial University, Harrogate, Tennessee, USA; ^2^Department of Cardiology, Methodist Le Bonheur Healthcare, Memphis, Tennessee, USA

**Keywords:** endovascular interventions, in-stent restenosis, peripheral arterial disease, stent graft, superficial femoral artery

## Abstract

Peripheral arterial disease (PAD) affects more than 230 million adults worldwide. Revascularization via angioplasty is a common method to manage stenosis in the superficial femoral artery (SFA). In-stent restenosis, however, is a common complication in endovascular interventions, especially in the SFA. Here, we present a case that involves recanalization of the SFA in a patient with a previously occluded stent and failed surgical revascularization. This patient initially presented with an occluded SFA which was stented. Four years later, the stent was reoccluded and surgical endarterectomy of the artery was performed with partial removal of the stent. Ten years later, the SFA is again occluded. Recanalization of the SFA using laser atherectomy and restenting of the occluded stent with GORE VIABAHN endoprosthesis was performed successfully. The combination of such methods is a suitable way to manage chronic lesions and minimize restenosis in patients with PAD.

## 1. Introduction

Peripheral arterial disease (PAD) affects more than 230 million adults worldwide [1]. Percutaneous intervention with stenting is a popular approach for treating PAD once conservative treatments fail. However, in-stent restenosis is a well-known and frequent complication of endovascular interventions. There are several options for revascularizing a previously stented artery. These primarily include performing percutaneous transluminal angioplasty (PTA) alone or with repeat stenting, drug-eluting balloon (DEB) angioplasty, or laser atherectomy [2]. Restenting can be performed with the use of bare-metal stents, drug-eluting stents, or stent grafts [2].

Excimer laser atherectomy (ELA) is a minimally invasive endovascular technique that aids in achieving revascularization of an artery through photoablation of plaque and hyperplastic tissue [3]. The EXCITE ISR trial, a randomized control study, that evaluated the efficacy of laser atherectomy using a Turbo-Elite laser catheter with PTA vs. PTA alone, revealed that ELA combined with PTA was superior to PTA alone for achieving revascularization in patients with PAD with femoropopliteal in-stent restenosis [2].

VIABAHN endoprosthesis (W.L. Gore & Associates, Flagstaff, Arizona, United States) with heparin bioactive surface is a stent graft that has been shown to be superior with higher patency rates when compared to standard balloon angioplasty and bare-metal stents [3, 4]. The heparin bonding of the graft acts to reduce thrombosis and neointimal hyperplasia, minimizing the need for reinterventions [4]. The RELINE study, a randomized controlled trial, comparing in-stent restenosis outcomes, showed that the GORE VIABAHN endoprosthesis performed significantly better when compared to a standard angioplasty balloon [3]. At 12 months, the primary patency rate was 28% with PTA alone and 74.8% in the GORE VIABAHN group [3]. The primary patency rate for the 24-month follow-up was 58.4% in the GORE VIABAHN group and 11.6% in the PTA group [5]. The VIASTAR trial, a randomized controlled trial, comparing the GORE VIABAHN endoprosthesis with bare-metal stents, revealed a significant improvement in patency at 1 year when using the GORE VIABAHN endoprosthesis (71% vs. 55%), with remarkable improvement in ankle-brachial index (ABI) as well [4]. In the SALVAGE trial, a prospective trial examining 27 patients with femoropopliteal in-stent restenosis, who were treated using ELA as an adjunctive treatment and the GORE VIABAHN endoprosthesis, reported a repeat revascularization rate of 17.4% [6].

Combining the evidence of these trials, for the patient presented in our case, we decided to perform laser atherectomy followed by the GORE VIABAHN endoprosthesis to restent the superficial femoral artery (SFA).

## 2. Case Presentation

A 67-year-old Caucasian male, former smoker with hypertension, hyperlipidemia, paroxysmal atrial fibrillation status post-Watchman device, COPD, and significant history of PAD previously followed by us, presented for a routine follow-up with complaints of intermittent right leg claudication. He had prior bilateral SFA stenting and endarterectomy of his right common femoral artery. ABI in the right leg was noted to be 0.75 and 1.11 in the left. Doppler ultrasound revealed monophasic waveform changes in the right SFA, popliteal, posterior tibial, and dorsalis pedis arteries. These findings along with diminished ABI suggested the presence of significant multilevel arterial occlusive disease of the right leg.

**Figure 1:**
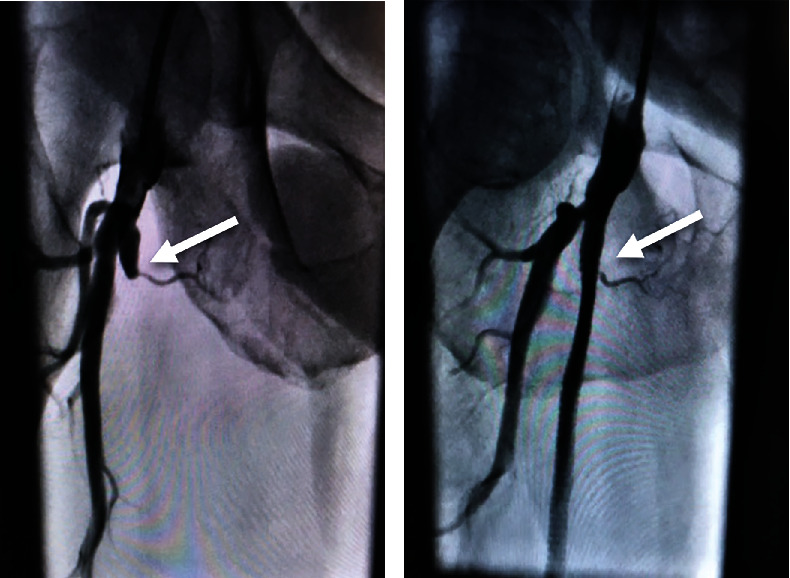
(a) 100% occlusion of the right SFA (white arrow). (b) Post–bare-metal stenting of the right SFA with complete return of flow (white arrow).

This was unfortunately the third time the patient had experienced claudication symptoms in his right leg with two previous failed interventions. This patient first presented in 2007 with progressive claudication symptoms of his right leg. Angiography was performed which confirmed 100% occlusion of the right SFA approximately 3 cm beyond its takeoff (Figure 1(a)). Intervention at that time involved implantation of two 7 × 150 metal stents, overlapping from about 2 cm distal to the occlusion to the very proximal portion of the SFA takeoff. These were then postdilated with a 5 × 120 balloon throughout the length of the stent. Angiography revealed that the original occlusion was reduced to essentially 0% (Figure 1(b)).

**Figure 2:**
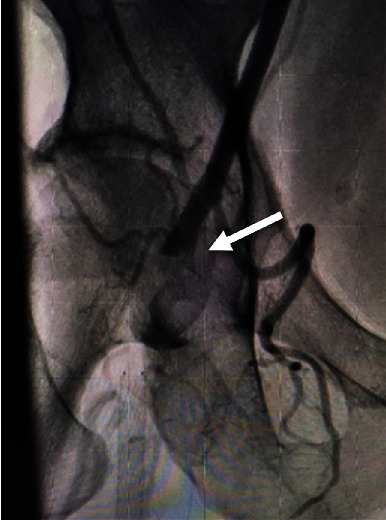
The right common femoral artery occluded above the branch point of the SFA (white arrow).

Four years later, this patient presented with recurring symptoms. Abdominal aortogram was performed and revealed that the right common femoral artery was occluded above the takeoff of the SFA, along with the SFA and deep femoral artery (Figure 2). The established plan then was to perform a femoral to popliteal bypass.

However, a change of surgical plan was made. Extensive thromboendarterectomy was carried out in the common femoral artery, deep femoral artery, and SFA, with the removal of two previous stents within the proximal SFA for a length of about 10 to 15 cm, with a partial stent remaining. Surgical thrombectomy was also performed, removing a large number of debris. After completion of the procedure, blood flow was restored to the right common femoral artery and all arteries distally. Intraoperative arteriography was carried out by placement of a 5-Fr sheath which revealed mild luminal irregularities but overall complete return of flow into the SFA, popliteal, posterior tibial, and fibular arteries.

The patient did well for 10 years before presenting with complaints of recurrence of claudication like symptoms. With reduced ABIs and monophasic waveforms, we decided to proceed with an abdominal aortogram with possible intervention.

**Figure 3:**
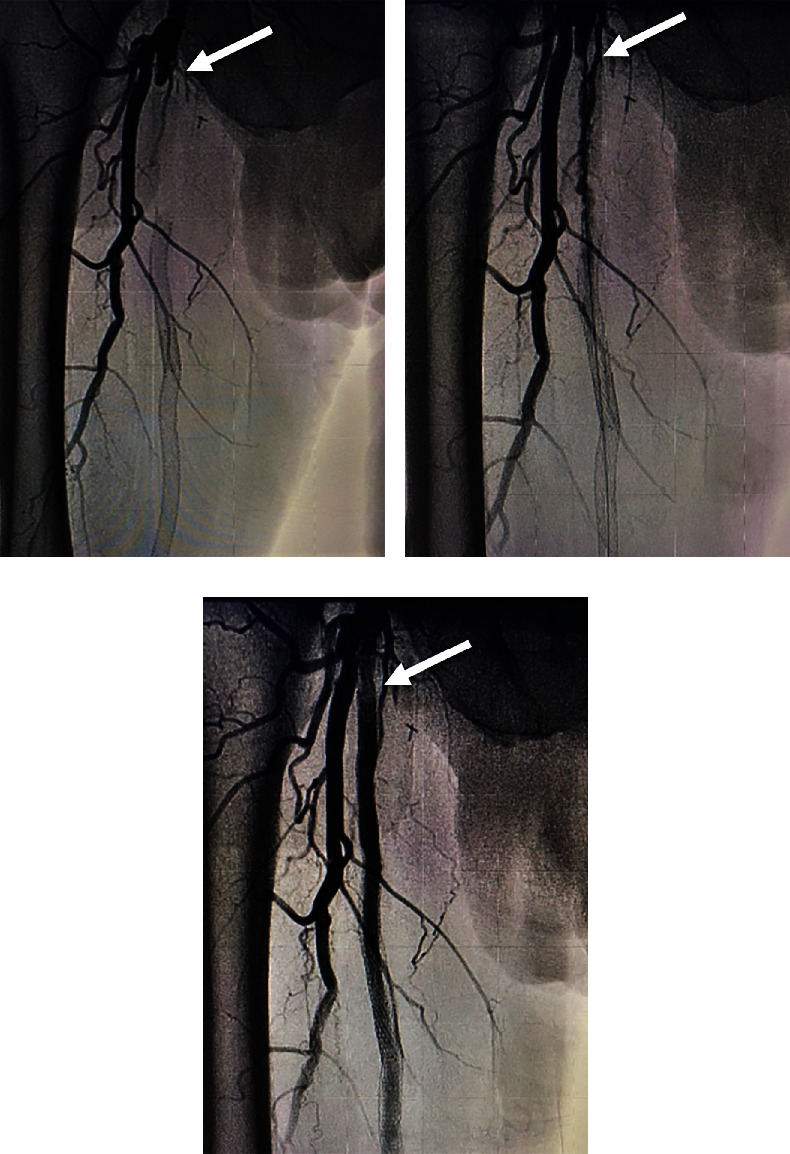
(a) 100% occlusion of the right SFA (white arrow). (b) Postlaser atherectomy of the right SFA (white arrow). (c) Successful GORE VIABAHN stent placement with complete return of flow (white arrow).

The patient was brought to the catheterization lab. Contralateral left groin access was obtained, and abdominal angiography followed by bilateral lower extremity angiography was performed. Abdominal aortogram revealed that the renal arteries, aorta, and iliac arteries were widely patent with some very mild luminal irregularities. Angiography of the right lower extremity revealed that the right SFA was 100% occluded at its takeoff. There were collaterals via the deep femoral artery reconstituting the SFA distally. The stent that was still present in the SFA began in the proximal to midsegment of the SFA and extended distally but was 100% occluded (Figure 3(a)). Below the knee, there was three-vessel runoff. At the conclusion of the diagnostic portion, we decided to proceed with intervention. Given the fact that the previous stent was occluded, and the patient had prior endovascular as well as surgical interventions, we decided to proceed with endovascular revascularization using laser atherectomy and restenting.

We exchanged for a 7-Fr Pinnacle destination sheath (Terumo Medical Corporation, Somerset, New Jersey, United States) which was advanced to the right common femoral artery. Using an angled glide catheter and a stiff-angled Glidewire (Terumo Medical Corporation, Somerset, New Jersey, United States), we were able to advance through the occluded portion of the SFA proximally and then advanced the wire into the occluded old stent within the SFA. We were then able to advance a stiff-angled Glidewire and catheter through the stent into the distal segment and then performed angiography confirming true lumen placement. At this point, we advanced a 018 wire through the glide catheter and then removed the catheter.

Laser atherectomy was performed through the occluded SFA using a 2.3 laser (Spectranetics Inc., Colorado Springs, Colorado, United States), establishing straight-line flow and then dilated with a 6-mm balloon (Figure 3(b)). This was followed by a placement of a 7 × 25- and 7 × 5-cm VIABAHN stent **(**W.L. Gore & Associates, Flagstaff, Arizona, United States**)** beginning just distal to the old stent and extending into the proximal SFA at the most normal segment of the vessel. The stent was then postdilated with a 6.0 balloon, and repeat angiography showed that the original occluded stent was now widely patent (Figure 3(c)). The patient tolerated the procedure well without complications.

The patient was started on dual antiplatelet therapy (DAPT), with aspirin and clopidogrel. This was continued for 12 months, after which aspirin was discontinued, as the patient endorsed developing gastrointestinal symptoms, thereafter continuing clopidogrel only.

ABI performed at 1-year follow-up was within normal limits (0.98 on the right lower extremity and 0.91 on the left lower extremity). The patient remains asymptomatic to date.

## 3. Discussion

The initial intervention in the SFA of our patient was performed using bare-metal stents. Since restenosis occurred in the common femoral artery, a relative “no-stent zone” [7], surgical intervention was recommended. A femoropopliteal bypass was initially planned. However, femoral endarterectomy with partial stent extraction was performed. This procedure was successful, with the femoral artery and following distal arteries remaining patent and therapeutically asymptomatic for 10 years. With the final reocclusion of the SFA and in-stent restenosis, revascularization with repeat stenting, using laser atherectomy for debulking, was planned and performed.

### 3.1. In-Stent Restenosis

In-stent restenosis is a common complication of endovascular interventions occurring due to excessive tissue proliferation within the lumen of the stent, also termed neointimal proliferation or hyperplasia [8]. The vascular process of restenosis is quite complex, with many contributing factors and mechanisms. Mechanical vessel injury due to stenting can lead to the migration of various cells, growth factors, and the production of an extracellular matrix. Endothelial disruption leads to the activation and adhesion of platelets, fibrin formation, and thrombosis [9, 10]. Proliferation of vascular smooth muscle cells, contributing to neointimal growth, continued inflammation, and vessel remodelling, leads to the overall narrowing of the vessel and the luminal diameter of the stent and surrounding area [11]. One classification system, developed by Tosaka et al. [12], categorizes in-stent restenosis based on its angiographic appearance: Class I or focal, Class II or diffuse, and Class III or totally occluded.

### 3.2. Management of In-Stent Restenosis

To minimize and manage in-stent restenosis, advancements with drug-eluting stents, DEBs, rotational and ELA, and endoprosthesis such as GORE VIABAHN offer favorable therapeutic options. A systematic review of four randomized controlled trials comparing various treatment strategies for in-stent restenosis revealed significantly higher freedom from target lesion revascularization (TLR) with ELA combined with standard balloon angioplasty, drug-coated balloon (DCB) angioplasty, and heparin-bonded GORE VIABAHN endoprosthesis, when compared to standard balloon angioplasty alone. However, no statistically significant differences were found when comparing between treatment strategies [13]. Hence, the optimal treatment strategy for managing in-stent restenosis in femoropopliteal lesions remains unclear [6, 13]. Currently, there are no established guidelines or recommendations for the appropriate management of in-stent restenosis [14]. The American College of Cardiology/American Heart Association's guidelines on the management of PAD provide no recommendations for specific endovascular therapy for in-stent restenosis, leaving it to the clinician's discretion based on the individual patient's clinical situation [15, 16].

Several studies have examined the use of DEB or DCB to manage in-stent restenosis. The FAIR trial, assessing DCB vs. a standard angioplasty balloon, with mean lesion length of 8.2 cm, revealed lower rate of recurrent in-stent stenosis at 6 months in DCB group when compared to standard angioplasty balloon and freedom from TLR of 90.8% in DCB group vs. 52.6% at 12 months [17]. The DEBATE-ISR trial which studied a paclitaxel-eluting balloon vs. standard angioplasty in patients with femoropopliteal in-stent restenosis revealed lower TLR in the DEB vs. standard balloon angioplasty at 1-year (13.6% vs. 31%, respectively); however, this became equivalent between the two groups at the 3-year follow-up (40% in DEB vs. 43% in standard balloon angioplasty group) [18, 19]. In addition, poor outcomes were seen in more complex lesions (Tosaka Classes II and III) [18, 19], a finding also seen in other similar studies [20]. The PACUBA trial revealed improved primary patency with DEB when compared to PTA (40.7% vs. 13.4%); however, no difference was noted in the secondary endpoints such as ABI, improvement in Rutherford classifications, and clinically driven TLR [21]. Although DCBs have shown promising results, these techniques have primarily shown benefit for shorter and less complex lesions, with decreasing long-term benefit, overall remaining an ineffective strategy to manage in-stent restenosis [14, 22].

Drug-eluting stents have also been studied for the treatment of in-stent restenosis, with promising results. The Zilver PTX study investigating the effectiveness of a drug-eluting stent in femoropopliteal in-stent restenosis revealed 1-year primary patency of 78.8% [23]. The postmarket surveillance study revealed 5-year rate of freedom from TLR of 73.4% [24]. Despite its effectiveness, currently, it remains unapproved for lesion lengths greater than 14 cm [25]. Stent grafts such as the GORE VIABAHN endoprosthesis are favorable especially for longer, diffuse lesions with favorable long-term patency rates regardless of lesion length [4, 10].

### 3.3. GORE VIABAHN Endoprosthesis

Currently, GORE VIABAHN endoprosthesis is the only stenting treatment approved by the Food and Drug Administration (FDA) for in-stent restenosis [25]. The advantage of GORE VIABAHN endoprosthesis is that due to its covered membrane, it minimizes the area of restenosis within the device lumen, limiting neointimal tissue growth. With potentially only two locations where restenosis can occur, the proximal and distal locations, the need for revascularization remains low. In addition, the expanded polytetrafluorethylene (ePTFE) covering of this stent reduces neointimal proliferation by forming a barrier and limiting smooth muscle cell growth. The addition of the heparin bioactive surface serves to reduce platelet formation, reducing the risk of thrombosis and neointimal hyperplasia, prolonging primary patency rates [3, 4]. As mentioned, GORE VIABAHN stent grafts have been shown to be superior particularly for longer lesions. In the RELINE study, comparing in-stent restenosis in GORE VIABAHN vs. standard angioplasty balloon, lesions stented with GORE VIABAHN exceeded 17 cm on average. The VIASTAR trial, comparing GORE VIABAHN to bare-metal stents, treated lesions greater than 20 cm, with a patency rate of 71.3% compared to 36.8% in bare-metal stents at 12 months [4]. In addition, the GORE VIABAHN stent graft is effective in treating occlusive lesions with long-term patency. A retrospective cohort study examining the patency of patients with occlusive lesions in the femoropopliteal artery treated with GORE VIABAHN stent graft revealed that primary patency at 1, 2, 3, and 4 years were 81.7%, 74.7%, 67.6%, and 58.9%, respectively, with secondary patency at 90.1% at 4 years [26].

The RELINE MAX clinical study, a prospective, single-arm postmarket study assessing the safety and effectiveness of the GORE VIABAHN endoprosthesis over 3 years, analyzed 86 patients within the United States and Europe with symptomatic PAD (Rutherford categories 2–5), at least 50% in-stent restenosis and/or occlusion within or adjacent to a previously implanted bare-metal stent in the SFA. It tested primary patency at 12 months, device- or procedure-related serious adverse events (SAEs) at 30 days, and freedom from TLR. The mean core laboratory reported lesion length was 12.4 ± 6.92 cm, with 81.9% of lesions being Tosaka Classes II and III. It was found that the procedural success was 98.8% with freedom from device-related SAEs which was 96.5% through 30 days. The primary patency rate at 1, 2, and 3 years was 74.7%, 55.9%, and 44.7%, respectively, and freedom from target revascularization at 1, 2, and 3 years was 84.8%, 74.6%, and 65%, respectively. In addition, 80.4% of patients had ≥ 1 Rutherford category improvement [25]. This study demonstrated that the GORE VIABAHN endoprosthesis is an effective way to treat long and complex lesions with favorable results through 3 years.

### 3.4. Laser Atherectomy

Revascularization with laser atherectomy has been shown to be beneficial for in-stent restenosis as an adjunctive treatment. The EXCITE trial, a multicenter, prospective, randomized controlled trial, tested mean lesions 19.6 ± 12.0 cm revealed that ELA plus PTA arm had higher procedural success (93.5% in combined group vs. 82.7% in PTA only group) and higher 6-month freedom from TLR (73.5% vs. 51.8% in PTA only group). Laser atherectomy functions by ablating hyperplastic tissue inside the stent [2]. This creates a pathway for balloon and stenting to be performed and decreases the stenotic area within the vessel. Using both techniques, laser atherectomy followed by stenting with a stent graft such as the GORE VIABAHN endoprosthesis offers an ideal option for in-stent restenotic lesions, especially for longer lesions in areas such as the SFA.

The benefit of using the complementary strategy of laser atherectomy and the use of a stent graft, such as GORE VIABAHN, has been shown in the SALVAGE trial [6]. This multicenter prospective trial involved 27 patients with femoropopliteal in-stent restenosis with mean lesions 20.7 cm ± 10.3 cm. All lesions were first treated with excimer laser and PTA prior to insertion of GORE VIABAHN endoprosthesis. The trial revealed no major adverse events at 30 days, and 12-month primary patency was 48%, with TLR of 17.4% [6].

The optimal method to manage in-stent restenosis remains uncertain, as no randomized controlled trials directly comparing the different methods currently exist. Considering the relatively limited clinical data for the management of in-stent restenosis, we opted to use laser atherectomy in conjunction with GORE VIABAHN stenting to maximize our odds of long-term patency.

Our case illustrates the successful management of a patient with complex PAD, utilizing a combination of laser atherectomy and the GORE VIABAHN endoprosthesis, leading to symptomatic relief and clinical patency for 12 months despite prior failed surgical and endovascular revascularizations. Maximal benefit for patients regarding any interventional procedure is of course achieved with proper modification of diet and lifestyle, cessation of smoking, a moderate exercise regimen, and guideline-directed medical therapy.

## 4. Conclusion

Revascularization via angioplasty is a common method to manage severe PAD. In-stent restenosis continues to be a common complication of endovascular procedures. Utilizing the GORE VIABAHN endoprosthesis proves to be a clinically favorable approach for addressing complex in-stent restenosis. ELA has shown to be beneficial as an adjunctive treatment in the management of in-stent restenosis. In our patient, laser atherectomy was first used to create a clear path to access the totally occluded lesion, to which a GORE VIABAHN stent graft was deployed. This combination of methods is a suitable way to manage such lesions and takes advantage of debulking and stenting to minimize restenosis with modalities that have been studied in randomized control trials.

## 5. Limitations

Case reports are prone to biases which can skew interpretations and limit generalizability. The main limitation of this case report is that it involves one patient with no control group. These biases emphasize the need for careful interpretation and consideration within the context of larger studies. Additionally, the lack of long-term follow-up data restricts the assessment of sustained efficacy and potential late complications of the treatment strategies.

## Data Availability

The studies mentioned in this paper can be accessed at the listed references.
